# In Vitro Evaluation of the Effects of Different Beverages on the Surface Microhardness of a Single-Shade Universal Composite

**DOI:** 10.7759/cureus.43669

**Published:** 2023-08-17

**Authors:** Ipsitha Vejendla, Hima Sandeep A, Pradeep S, Sahil Choudhari

**Affiliations:** 1 Conservative Dentistry and Endodontics, Saveetha Dental College and Hospitals, Saveetha Institute of Medical and Technical Sciences, Saveetha University, Chennai, IND

**Keywords:** single shade composite, vickers microhardness, beverages, surface microhardness, composite resin

## Abstract

Aim: The aim of this in vitro study was to analyze the influence of various beverages on the surface microhardness of a single-shade composite resin.

Materials and methods: Seventy-five Omnichroma composite discs were fabricated and divided into five subgroups according to the beverages. The groups were Group A: tea; Group B: coffee; Group C: beer; Group D: whisky; and Group E: artificial saliva. The samples were immersed in their respective beverages for 15 minutes per day for 15 days. The Vickers microhardness values were taken before and after the immersion of the composite disc samples in their respective beverages. A one-way analysis of variance (ANOVA) test was conducted using IBM SPSS Statistics software version 23 (IBM Corp., Armonk, New York, USA), with a statistical significance level of 5%.

Results: It was observed that there was a reduction in the surface microhardness of the composite resin after immersion in most of the solutions. An increase in the mean percentage change of surface microhardness was observed in the beer group (29%; mean difference = 16.52±12.05), followed by the coffee group (27.2%; mean difference = 15.51±9.28). The least mean percentage change in microhardness was noted in group E, artificial saliva (8.5%; mean difference = 4.81±3.04).

Conclusion: The surface microhardness of composite resin can be influenced by the type of beverages used for immersion and the duration of immersion. However, it is important to note that the interaction between composite resin and various beverages is influenced by a complex interplay of multiple variables.

## Introduction

The rising popularity of resin-based composite dental materials can be attributed to the growing demand for aesthetically pleasing, tooth-colored, and mercury-free restorations. However, there are several factors that may hinder their clinical efficacy [1]. The mechanical and physical properties of composite resin restorations, including durability, hardness, resistance to abrasion, surface smoothness, prevention of secondary caries, susceptibility to microleakage, plaque accumulation, the overall appearance of the restoration, and patient satisfaction, all contribute to their ultimate success [1,2].

The basic qualities of composite materials, which are used in dental fillings, might alter over time due to frequent exposure to noxious elements (mechanical, thermal, or chemical) in the oral cavity [2,3]. Chemical influences could be external, such as acidic foods and chlorinated water, or internal, such as gastric acids in frequent vomiting. These acids have the potential to erode composite materials and the hard tissues of teeth, thereby affecting the quality of restoration [2].

Most of the time, the wearing of the dental restoration, along with the tooth structure and subsequent discoloration, causes the need for a change in the restoration. Consumption of certain beverages like tea, coffee, soft drinks, and alcoholic beverages may impact the quality of the restoration and affect its surface hardness at different rates [3]. The effect of these beverages on the quality of the restoration depends on the chemical composition of the restorative material. The wear and discoloration of restorative materials are known to be influenced by the size, concentration, and resin formulation of the filler particles [4]. Previous literature reported that the amount of spacing between the filler particles that provide protection from food bolus determines the wear resistance of composites in the oral cavity, thereby stating that the smaller filler particles occupying the inter-particle space in the composite improve its wear resistance [5, 6].

Clinicians often prefer restorative materials and techniques that allow for simplified restorative procedures, aiming to reduce chair time and minimize technique sensitivity. One particular challenge in restorative dentistry is shade selection, as it can be difficult and is influenced by the operator's skill and environmental conditions [7,8]. To address this issue and make shade selection more convenient, universal composites have been developed. These composites, exemplified by products like Omnichroma, are single-shade materials that claim to match all 16 VITA Classical shades, ranging from A1-D4®, enabling a shade match for various tooth colors [9]. This advancement offers greater versatility and efficiency in achieving aesthetically pleasing restorations.

Dental restorations, especially resin-based composites, are commonly used in dental practice. Understanding how various beverages can impact the microhardness of these restorations is clinically relevant. It provides insights into potential challenges that dentists and patients may face when exposed to certain everyday beverages and helps in making informed decisions about the choice of restorative materials for specific patients. This study evaluates a single-shade universal composite material claiming to match various tooth colors. Understanding how this material interacts with different beverages can help assess its performance and validate its claims. Such research contributes to the advancement of dental materials and encourages the development of more durable and aesthetically pleasing options for dental restorations. Overall, conducting this study helps bridge the gap in knowledge regarding the impact of beverages on composite resin restorations and provides essential information for better clinical decision-making, improved patient outcomes, and the advancement of dental materials.

This study has been conducted to analyze the interaction of the above-mentioned composite resin with various beverages and determine the resultant surface characteristics. The aim of this investigation was to determine how various beverages affect the microhardness of a single-shade universal resin composite material. The null hypothesis was formulated, stating that the evaluated beverages would have no effect on the microhardness of the tested composite material.

## Materials and methods

This in-vitro study was performed at Saveetha Dental College and Hospital, Chennai, India, in January 2022.

Sample preparation

The sample size of the current study was calculated using G*Power (Heinrich Heine University Düsseldorf, Düsseldorf, Germany) based on an in vitro assessment by Tanthanuch et al. with an effect size of 0.52, significance of 5%, and power of the study of 95% [10,11].

The Palfique Omnichroma (Tokuyama Dental Corporation, Tokyo, Japan) single-shade universal composite material was the composite resin that was evaluated in this investigation. The manufacturer's details and composition of the evaluated resin composite are described in Table 1.

**Table 1:** The composition of composite material evaluated in the study UDMA: urethane dimethacrylate, TEGDMA: triethylene glycol dimethacrylate

Material	Manufacturer details	Type of material	Average particle size	Matrix	Filler components	Filler content
Palfique Omnichroma	Tokuyama Dental, Tokyo, Japan	Supra-nano spherical composite	About 200nm	TEGDMA, UDMA	Spherical-shaped, uniformly sized supra-nano spherical filler (260 nm spherical SiO2-ZrO2)	79 wt% (68 vol%)

Seventy-five composite disc-shaped samples were prepared using a stainless steel mold with dimensions of 10 mm in diameter and 2 mm in thickness. The resin composite was placed into the mold and sandwiched between the translucent mylar strips and two thin glass slides. The samples were then light-cured for 40 seconds using a light-emitting diode (LED) unit (2300 mW/cmÂ², Woodpecker O-Light 1 Second Curing Light Unit, DTE Woodpecker, China). Following that, the samples were polished using the Super-Snap polishing system (Shofu Inc., Kyoto, Japan) and polishing discs as per the manufacturer's instructions. After sample preparation, they were stored in distilled water for 24 hours in order to complete their polymerization process and rehydration.

The resin-based composite material samples were randomly divided into five groups of 15 samples each, based on the beverages used. The groups were:

Group A: Tea (Brooke Bond Taj Mahal, Hindustan Unilever Ltd., Mumbai, India)

Group B: Coffee (Bru Instant, Hindustan Unilever Ltd., Mumbai, India)

Group C: Beer (Kingfisher Strong Beer, United Breweries Group, Bangalore, India)

Group D: Whisky (Royal Challenge Premium Whisky, United Spirits Ltd., Bangalore, India)

Group E: Artificial Saliva (Wet Mouth, ICPA Health Products Ltd., Mumbai, India)

After 24 hours of storage in distilled water, the samples were blotted dry with tissue paper. Baseline values of the surface microhardness of each sample were taken for each group.

Sample immersion protocol

The immersion protocol was followed for 15 days, based on the assessment by Barve et al. [12]. The beverage sample was changed every time, and each group's samples were submerged in their respective beverage for 15 minutes daily. Each sample was individually stored in distilled water while not immersed in its respective evaluating beverage. The surface microhardness assessment was done after 15 days.

Microhardness evaluation

The microhardness evaluation was performed on the surface of the composite resin samples using a Vickers indenter (HMV-G31DT Micro Vickers Hardness Tester, Shimadzu, Kyoto, Japan). Three indentations were made for each specimen over the course of 15 seconds with a 50-gram weight [12]. The relative percentage change in microhardness is analyzed using the following formula:

(Microhardness Before - Microhardness After) X 100 / Microhardness Before

Statistical analysis

The statistical analysis was performed using the IBM Statistical Package for Social Sciences software (SPSS Statistics; version 23, IBM Corp., Armonk, New York, USA). The Shapiro-Wilk test was performed to determine the distribution of values achieved for surface microhardness assessment. The surface microhardness values were tested for significance using a one-way analysis of variance (ANOVA) and Tukey’s post hoc analysis. All the statistical tests used in the present study consider a p-value less than 0.05 to be significant.

## Results

A single-shade composite resin, Omnichroma, was used to assess its interaction with five different immersion solutions. The surface microhardness assessment was done based on the Vickers indentation method.

**Figure 1:**
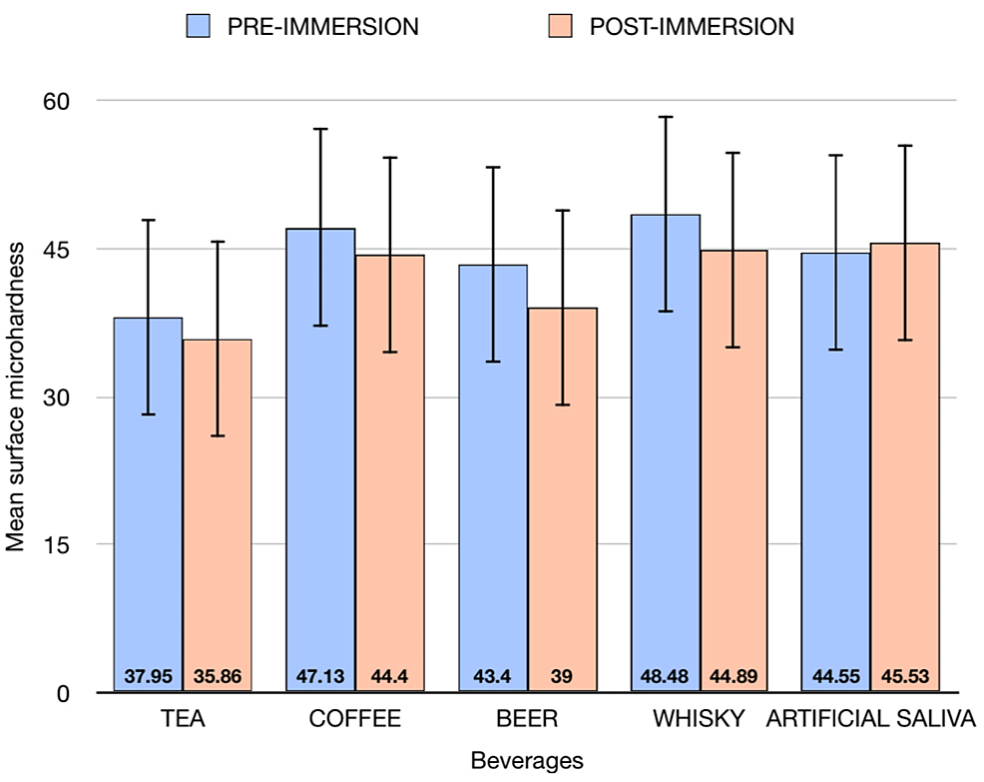
A graph depicting the baseline and 15-day surface microhardness values

A 15-day immersion protocol has been followed after recording the baseline microhardness values. The mean surface microhardness, along with standard deviation values at baseline and post-immersion (15th day) of the evaluated composite discs, is noted in Table 2 and represented in Figure 1.

**Table 2:** Mean and standard deviation values of surface microhardness of the assessed beverage groups

Beverage Groups	Group A (Tea)	Group B (Coffee)	Group C (Beer)	Group D (Whisky)	Group E (Artificial saliva)
Before immersion (baseline)	37.94 ± 4.85	47.12 ± 5.65	43.39 ± 5.72	48.48 ± 6.05	44.55 ± 4.42
After immersion (15 days)	35.86 ± 5.07	44.4 ± 3.45	38.99 ± 6.44	44.89 ± 6	45.53 ± 3.9

The mean percentage change in surface microhardness of the different groups and the inter-group comparison of microhardness change in different groups are depicted in Tables 3-4, respectively.

**Table 3:** Mean percentage change calculated for the assessed solution groups: as percentage and (Mean±SD)

Beverage Groups	Group A (Tea)	Group B (Coffee)	Group C (Beer)	Group D (Whisky)	Group E (Artificial Saliva)
Mean percentage change in microhardness	20.8% (11.83±6.66)	27.2% (15.51±9.28)	29% (16.52±12.05)	14.5% (8.28±3.16)	8.5% (4.81±3.04)

**Table 4:** Inter-group comparison of evaluated beverages in terms of percentage change in surface microhardness using Tukey’s post hoc test * indicates a statistically significant difference (p≤0.05)

Beverages	Beverages	Mean Difference	p-value
Tea	Coffee	3.681	0.723
	Beer	4.691	0.508
	Whisky	3.557	0.747
	Artificial saliva	7.021	0.132
Coffee	Tea	3.681	0.723
	Beer	1.009	0.997
	Whisky	7.238	0.113
	Artificial saliva	10.702	0.005*
Beer	Tea	4.691	0.508
	Coffee	1.009	0.997
	Whisky	8.248	0.050*
	Artificial saliva	11.711	0.002*
Whisky	Tea	3.557	0.747
	Coffee	7.238	0.113
	Beer	8.248	0.050*
	Artificial saliva	3.463	0.766
Artificial saliva	Tea	7.021	0.132
	Coffee	10.702	0.005*
	Beer	11.711	0.002*
	Whisky	3.463	0.766

**Figure 2:**
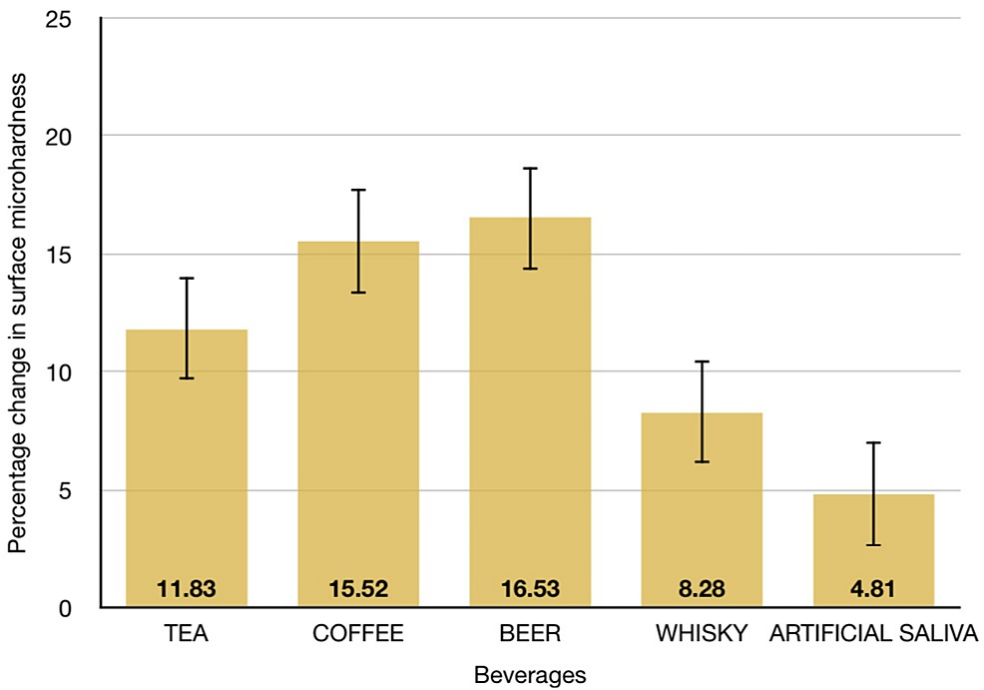
A graph depicting the percentage change calculated for the assessed solution groups

Figure 2 represents the percentage change calculated for the assessed solution groups.

The percentage change in the microhardness was greatest when the samples were subjected to beer (Group C; mean difference = 16.52±12.05), followed by coffee (Group B; mean difference = 15.51±9.28), and the least change was reportedly seen in artificial saliva (Group E; mean difference = 4.81±3.04).

**Figure 3:**
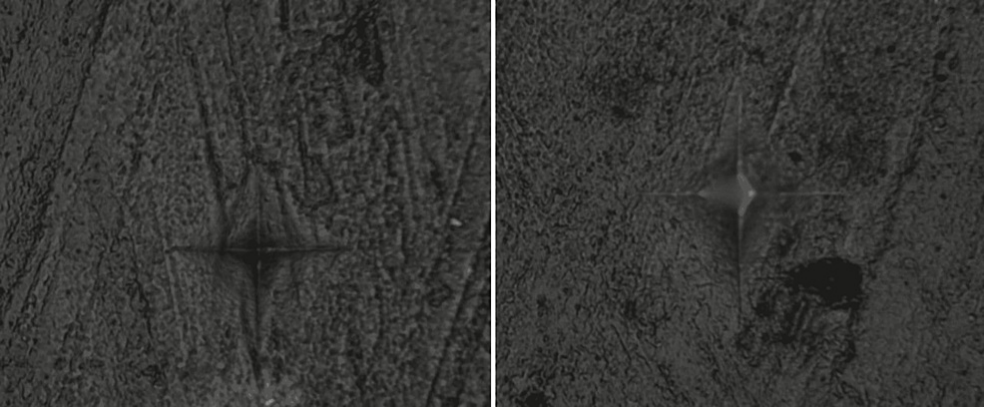
Photomicrographs of microhardness assessment in Group C (Beer): baseline (left) and after 15 days of immersion (right)

**Figure 4:**
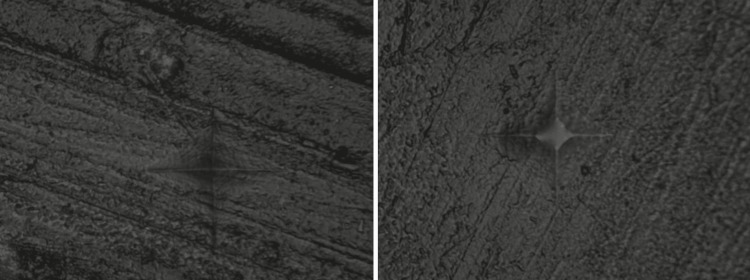
Photomicrographs of microhardness assessment in Group B (coffee): baseline (left) and after 15 days of immersion (right)

**Figure 5:**
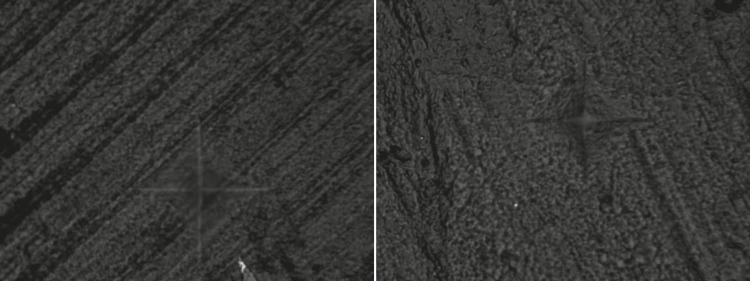
Photomicrographs of microhardness assessment in Group E (artificial saliva): baseline (left) and after 15 days of immersion (right)

The findings showed that Groups C (beer), B (coffee), and A (tea) presented the greatest change in surface microhardness. Following the 15-day immersion protocol, Group E (artificial saliva) exhibited the least change in microhardness values. Figures 3-5 display the photomicrographs of the Vickers indentation microhardness assessment in the beer, coffee, and artificial saliva groups, respectively.

## Discussion

A significant increase in the clinical usage of resin-based restorative materials has been observed over the past few years, owing to their improved formulations, ease of handling, and excellent aesthetic and bonding properties [13,14]. Ideally, restorative materials must have long-term consciousness and the ability to not be greatly influenced by the exposed environment in order to be clinically effective [15]. It is important to consider that the oral cavity is a multifaceted environment where restorative materials are constantly exposed to saliva and affected by systemic and dietary factors [14]. The characteristics of the restorative material, including its clinical effectiveness and long-term performance, are influenced by various chemical and thermal variables. Among these variables, chemical factors play a crucial role in the surface degradation of composite resins [16], which in turn can lead to alterations in microhardness [2,17].

The current in vitro study evaluates the effect of various beverages on the surface microhardness of a single-shade universal resin composite. Tokuyama Dental America has unveiled the Smart Chromatic Technology in the evaluated dental composite, Omnichroma. This innovation uses uniform spherical silica and zirconia filler particles to regulate light transmission in the red-to-yellow color range. As a result, Omnichroma seamlessly blends with surrounding teeth for a natural look.

It is essential to determine and understand the mechanical properties of composite resins to comprehend and foresee their clinical behavior and durability [18]. Based on the results obtained, there was no significant difference seen in the change in surface microhardness of the resin composite (p>0.05), thereby accepting the null hypothesis proposed. The percentage change in the microhardness was greatest when the samples were subjected to the beer group, followed by the coffee group, and the least change was reportedly seen in the artificial saliva group.

The hardness of the restorative material is regarded as a crucial characteristic as it corresponds favorably with the compressive strength, resistance to intraoral components, and conversion degree of the material [13,19]. Declining surface microhardness values result in poor wear resistance and a propensity for scratching, which may further compromise the longevity of the restoration [20]. Along with the consideration of surface microhardness properties in the clinical application of composite restorations, other physicomechanical characteristics, such as flexural strength, wear resistance, the degree of conversion, and color stability, should also be taken into consideration when making a choice of composite resin for tooth restoration, particularly in high-stress locations [21].

The current investigation observed that immersion of the assessed resin composite in beer caused the maximum change in surface microhardness. This may be attributed to the absorption of alcohol molecules by the resin matrix, leading to a softening effect on the surfaces of the resin and consequently impacting the overall integrity of the restoration [22].

The study findings indicate that there is a decrease in the microhardness of the evaluated composite resin upon interaction with coffee. Despite having a pH close to seven, the water present in coffee gets absorbed by the resin matrix, causing disintegration of the restorative material. This, in turn, affects the coupling agents, leading to hydrolysis and weakening of the chemical bond between the filler particles and resin matrix. The dislodgment of filler particles from the material's outer surface results in surface roughness and a decrease in hardness [23]. Furthermore, the degradation of the resin-filler interface and the impact on the resin matrix and inorganic fillers can contribute to the observed reduction in surface hardness [24].

Depending on the nature of the composite resin, the surface characteristics of the resin will vary [25]. The main limitation of this study was that the complex oral environment was not accurately replicated, and the effects of temperature fluctuations and pH changes were not taken into account. Consumption of beverages can, however, have an impact on the oral environment, which could lead to a variety of consequences for the examined composite resins. The behavior of these beverages may also be hindered by the impact of other elements pertaining to the individual's oral cavity. Although the study highlights the importance of selecting appropriate restorative materials based on the specific clinical situation and patient factors, it is important to note that this study was conducted in an in vitro setting, and further research is needed to validate these findings in clinical settings and consider the influence of dietary and oral hygiene factors.

## Conclusions

The current study investigated the surface microhardness of a single-shade universal composite resin, and the findings revealed no statistically significant difference in surface microhardness following immersion in the respective solution groups. Within the limitations of the study, the current in-vitro assessment leads to the following conclusions: All the solutions had an impact on the change in surface microhardness of the resin composite; immersion of this single-shade resin composite in beer for 15 days had an aggressive effect on surface wear characteristics when compared to other solutions evaluated; and the maximum change in surface microhardness upon immersion of the evaluated composite resin for 15 days was observed in Group C (beer) samples, followed by Group B (whisky). The current assessment suggests that the chemical composition of beverages can impact the surface microhardness of resin composites, with alcohol absorption and water absorption playing significant roles that may alter the clinical behavior and durability of the resin material.

## References

[1] Chesterman J, Jowett A, Gallacher A, Nixon P (2017). Bulk-fill resin-based composite restorative materials: a review. Br Dent J.

[2] Szalewski L, Wójcik D, Bogucki M, Szkutnik J, Różyło-Kalinowska I (2021). The influence of popular beverages on mechanical properties of composite resins. Materials (Basel).

[3] Vogel RI (1975). Intrinsic and extrinsic discoloration of the dentition (a literature review). J Oral Med.

[4] Torii Y, Itou K, Itota T, Hama K, Konishi N, Nagamine M, Inoue K (1999). Influence of filler content and gap dimension on wear resistance of resin composite luting cements around a CAD/CAM ceramic inlay restoration. Dent Mater J.

[5] Bayne SC, Taylor DF, Heymann HO (1992). Protection hypothesis for composite wear. Dent Mater.

[6] Söderholm KJ, Richards ND (1998). Wear resistance of composites: a solved problem?. Gen Dent.

[7] Pereira Sanchez N, Powers JM, Paravina RD (2019). Instrumental and visual evaluation of the color adjustment potential of resin composites. J Esthet Restor Dent.

[8] Trifkovic B, Powers JM, Paravina RD (2018). Color adjustment potential of resin composites. Clin Oral Investig.

[9] Iyer RS, Babani VR, Yaman P, Dennison J (2021). Color match using instrumental and visual methods for single, group, and multi-shade composite resins. J Esthet Restor Dent.

[10] Kang H (2021). Sample size determination and power analysis using the G*Power software. J Educ Eval Health Prof.

[11] Tanthanuch S, Kukiattrakoon B, Siriporananon C, Ornprasert N, Mettasitthikorn W, Likhitpreeda S, Waewsanga S (2014). The effect of different beverages on surface hardness of nanohybrid resin composite and giomer. J Conserv Dent.

[12] Barve D, Dave P, Gulve M, Saquib S, Das G, Sibghatullah M, Chaturvedi S (2021). Assessment of microhardness and color stability of micro-hybrid and nano-filled composite resins. Niger J Clin Pract.

[13] Badra VV, Faraoni JJ, Ramos RP, Palma-Dibb RG (2005). Influence of different beverages on the microhardness and surface roughness of resin composites. Oper Dent.

[14] Erdemir U, Yildiz E, Eren MM, Ozel S (2013). Surface hardness evaluation of different composite resin materials: influence of sports and energy drinks immersion after a short-term period. J Appl Oral Sci.

[15] Yap AU, Tan SH, Wee SS, Lee CW, Lim EL, Zeng KY (2001). Chemical degradation of composite restoratives. J Oral Rehabil.

[16] Yap AU, Wattanapayungkul P, Chung SM (2003). Influence of the polymerization process on composite resistance to chemical degradation by food-simulating liquids. Oper Dent.

[17] Lee YK, Lu H, Oguri M, Powers JM (2005). Changes in gloss after simulated generalized wear of composite resins. J Prosthet Dent.

[18] Heintze SD, Ilie N, Hickel R, Reis A, Loguercio A, Rousson V (2017). Laboratory mechanical parameters of composite resins and their relation to fractures and wear in clinical trials- a systematic review. Dent Mater.

[19] Uhl A, Mills RW, Jandt KD (2003). Photoinitiator dependent composite depth of cure and Knoop hardness with halogen and LED light curing units. Biomaterials.

[20] Say EC, Civelek A, Nobecourt A, Ersoy M, Guleryuz C (2003). Wear and microhardness of different resin composite materials. Oper Dent.

[21] Knobloch L, Kerby RE, Clelland N, Lee J (2004). Hardness and degree of conversion of posterior packable composites. Oper Dent.

[22] Lepri CP, Palma-Dibb RG (2012). Surface roughness and color change of a composite: influence of beverages and brushing. Dent Mater J.

[23] Santos C, Clarke RL, Braden M, Guitian F, Davy KW (2002). Water absorption characteristics of dental composites incorporating hydroxyapatite filler. Biomaterials.

[24] EL-Sharkawy FM, Zaghloul NM, Ell-kappaney AM (2012). Effect of water absorption on color stability of different resin based restorative materials in vitro study. Int J Compos Mater.

[25] Yazici AR, Tuncer D, Antonson S, Onen A, Kilinc E (2010). Effects of delayed finishing/polishing on surface roughness, hardness and gloss of tooth-coloured restorative materials. Eur J Dent.

